# Evaluating the Clinical Feasibility of an Artificial Intelligence–Powered, Web-Based Clinical Decision Support System for the Treatment of Depression in Adults: Longitudinal Feasibility Study

**DOI:** 10.2196/31862

**Published:** 2021-10-25

**Authors:** Christina Popescu, Grace Golden, David Benrimoh, Myriam Tanguay-Sela, Dominique Slowey, Eryn Lundrigan, Jérôme Williams, Bennet Desormeau, Divyesh Kardani, Tamara Perez, Colleen Rollins, Sonia Israel, Kelly Perlman, Caitrin Armstrong, Jacob Baxter, Kate Whitmore, Marie-Jeanne Fradette, Kaelan Felcarek-Hope, Ghassen Soufi, Robert Fratila, Joseph Mehltretter, Karl Looper, Warren Steiner, Soham Rej, Jordan F Karp, Katherine Heller, Sagar V Parikh, Rebecca McGuire-Snieckus, Manuela Ferrari, Howard Margolese, Gustavo Turecki

**Affiliations:** 1 Aifred Health Inc. Montreal, QC Canada; 2 University of Waterloo Waterloo, ON Canada; 3 McGill University Montreal, QC Canada; 4 University of Cambridge London United Kingdom; 5 University of Arizona Tucson, AZ United States; 6 Duke University Durham, NC United States; 7 University of Michigan Ann Arbor, MI United States; 8 Barts and the London School of Medicine London United Kingdom; 9 Douglas Mental Health University Institute McGill University Montreal, QC Canada

**Keywords:** clinical decision support system, major depressive disorder, artificial intelligence, feasibility, usability, mobile phone

## Abstract

**Background:**

Approximately two-thirds of patients with major depressive disorder do not achieve remission during their first treatment. There has been increasing interest in the use of digital, artificial intelligence–powered clinical decision support systems (CDSSs) to assist physicians in their treatment selection and management, improving the personalization and use of best practices such as measurement-based care. Previous literature shows that for digital mental health tools to be successful, the tool must be easy for patients and physicians to use and feasible within existing clinical workflows.

**Objective:**

This study aims to examine the feasibility of an artificial intelligence–powered CDSS, which combines the operationalized 2016 Canadian Network for Mood and Anxiety Treatments guidelines with a neural network–based individualized treatment remission prediction.

**Methods:**

Owing to the COVID-19 pandemic, the study was adapted to be completed entirely remotely. A total of 7 physicians recruited outpatients diagnosed with major depressive disorder according to the *Diagnostic and Statistical Manual of Mental Disorders*, *Fifth Edition* criteria. Patients completed a minimum of one visit without the CDSS (baseline) and 2 subsequent visits where the CDSS was used by the physician (visits 1 and 2). The primary outcome of interest was change in appointment length after the introduction of the CDSS as a proxy for feasibility. Feasibility and acceptability data were collected through self-report questionnaires and semistructured interviews.

**Results:**

Data were collected between January and November 2020. A total of 17 patients were enrolled in the study; of the 17 patients, 14 (82%) completed the study. There was no significant difference in appointment length between visits (introduction of the tool did not increase appointment length; *F*_2,24_=0.805; mean squared error 58.08; *P*=.46). In total, 92% (12/13) of patients and 71% (5/7) of physicians felt that the tool was easy to use; 62% (8/13) of patients and 71% (5/7) of physicians rated that they trusted the CDSS. Of the 13 patients, 6 (46%) felt that the patient-clinician relationship significantly or somewhat improved, whereas 7 (54%) felt that it did not change.

**Conclusions:**

Our findings confirm that the integration of the tool does not significantly increase appointment length and suggest that the CDSS is easy to use and may have positive effects on the patient-physician relationship for some patients. The CDSS is feasible and ready for effectiveness studies.

**Trial Registration:**

ClinicalTrials.gov NCT04061642; http://clinicaltrials.gov/ct2/show/NCT04061642

## Introduction

### Background

Clinical decision support systems (CDSSs) consolidate large quantities of clinical information to provide clinicians with the necessary data to support medical decision-making and assist with managing treatment protocols [1-3]. An emerging focus of medical informatics is the improvement of patient care through data-driven, patient-centered decision support systems. Artificial intelligence (AI) algorithms are increasingly being integrated into CDSSs, permitting predictive analytics to be used by clinicians as part of routine practice [3]. The overarching objective of these systems is to improve medical decision-making using a data-driven approach. However, although much has been written about machine learning techniques [4,5] that underpin the technical advancements that make these systems possible, comparatively less focus has been placed on the usability and feasibility of these kinds of systems in medicine in general and in mental health treatment in particular. In this paper, we discuss a feasibility study of a novel AI-powered CDSS aimed at improving the treatment of depression.

Feasibility and ease of use are major concerns as they roughly equate to the tolerability of drug treatment; with similar impact—much like a medication—a digital tool can only have a positive impact if patients (and, in this case, clinicians) use it and continue to use it. A recent meta-analysis of randomized controlled trials aimed to establish the dropout rates of studies on medical smartphone apps tracking depressive symptoms [6]. The analysis found that apps for depressive symptom tracking had a dropout rate of approximately 50% when accounting for bias. Despite this high dropout rate, there is some knowledge about how to reduce dropouts. For example, researchers found that the dropout rate was significantly lower—as low as 12%—in apps offering human feedback and in-app mood monitoring [6]. In addition, previous decision support systems have demonstrated the need for a tool that provides real-time utility [7] and the ability to personalize treatment choices and differentiate between medications [8] in a quantifiable manner [9] and incorporates clinical practice guidelines [10].

Only one-third of patients with depression who receive treatment will achieve remission during the first treatment course; most experience multiple treatment trials before entering remission [11]. Clinicians are faced with a wide range of treatment options, in combination with associated guidelines, to manage the selected treatments. However, there are no easily accessible point-of-care tools available to aid in the optimization of treatment success and minimize the time to remission. Furthermore, treatments are essentially equally effective at the population level; as such, to improve outcomes, treatment selection must address the individual’s specific characteristics [10,12]. As such, there is a clear need for improved and personalized decision support for mental health care [13].

### The Aifred CDSS

Aifred is a CDSS that uses AI to assist clinicians in selecting treatments for major depressive disorder (MDD). The tool incorporates a deep-learning model that was validated and trained on clinical and demographic baseline data to support treatment selection by providing individualized probabilities of remission for specific treatment options. Please see the study by Benrimoh et al [10] for a description of the tool, and the studies by Mehltretter et al [4,14] for a description of the machine learning model and model training and validation methodology. Clinicians accessing the app first see their patient’s self-report questionnaire scores and can examine these through graphs showing trends over time or at the individual item level. They can then select the clinical algorithm, which is an operationalized version of the Canadian Network for Mood and Anxiety Treatments (CANMAT) 2016 guidelines for the treatment of depression [15]. These operationalized guidelines function in an entirely rule-based manner and take in patient depression scores at baseline and at subsequent visits to determine if patients have achieved early response or remission (based on guideline-informed criteria and questionnaire remission thresholds, respectively) and provide guideline-appropriate information at each patient visit. At all times, clinicians remain in control of clinical decisions and may select treatments or modify treatments as they feel are clinically indicated; there were no automated clinical decisions in this study. The AI aspect of the CDSS is specifically meant to assist clinicians with treatment choices. It is directly integrated into the operationalized CANMAT guidelines, with personalized predicted remission probabilities for individual treatments presented within the guideline module when antidepressant treatments are being chosen. This occurs in the following manner: whenever first-line treatment options are presented in the clinical algorithm, the AI model will provide predictions in the form of remission probabilities for a number of antidepressant medications based on a mix of symptom and demographic information provided by patients at baseline. These remission probability predictions are generated by the AI model using questionnaires responded to early in treatment and are meant to help guide initial treatment selection or change; further treatment management support (eg, information about switching, dose adjustment, or augmentation options if patients do not show improvement with treatment) is provided longitudinally by the rule-based CANMAT algorithm. The AI is designed to support clinicians by considering complex interactions among multiple patient clinical, social, and demographic variables to help personalize treatment to improve upon a trial-and-error treatment approach and reduce the number of failed treatment trials [10]. To summarize, the app assists clinicians in providing measurement-based, treatment algorithm–guided, and AI-personalized care.

Providing patients with the ability to monitor their own mental health symptoms has attracted a great deal of interest across all ages [16]. Patients also have access to their own version of the Aifred app wherein they respond to questionnaires and can view their active and past treatments, as well as their symptoms graphed over time. This availability of data to both physicians and patients is intended to empower patients, enrich conversations, and facilitate shared decision-making [10].

### Study Aims

Following a previous simulation center study [10] and ahead of larger clinical trials aimed at assessing safety and effectiveness, we decided to conduct a feasibility study aimed at exploring the feasibility of the CDSS in a real clinical setting and to assess its longitudinal impact on the patient-clinician relationship. This study has 4 aims:

1. To assess the feasibility of the CDSS for use in clinical practice
2. To assess physician and patient trust in the CDSS and its effect on the clinician-patient relationship
3. To assess the usability of the CDSS and study software and to ensure that major limitations are identified and rectified before clinical trials
4. To assess engagement with the app

One key metric brought up by clinicians interviewed during initial stakeholder conversations was appointment length; clinicians are increasingly required to interact with time-consuming digital systems, and the fear of yet another system adding time to assessments is a reasonable one [17]. We aim to measure appointment length as our primary outcome, as a key numerical proxy for real-world feasibility.

## Methods

### Overview

The study was approved by the research ethics board of the Douglas Mental Health University Institute (identifier: NCT04061642). All participants provided written informed consent to participate. The study was conducted according to the ethical principles stated in the Tri-Council Policy Statement on the Ethical Conduct for Research Involving Humans [18].

This was a single-arm, naturalistic follow-up study aimed at assessing software usability and acceptability conducted between January and November 2020. This study was not designed to assess the clinical effectiveness of the tool, which will be the focus of an upcoming clinical trial. It is important to note that physicians were provided access to the tool but were free to use the tool and its AI predictions or ignore it.

The study sample included 2 population groups: (1) physicians, including family physicians and psychiatrists and (2) patients of these physicians. The recruitment target was 10 physicians and 3-4 patients per physician (30-40 patients in total).

Physicians were recruited via recruitment email and direct contact by the study personnel. The sites consisted of university hospitals, primary care clinics, and psychiatric clinics in the Canadian province of Québec. Eligible physicians were family physicians or psychiatrists treating patients with depression on at least a monthly basis. Physicians who met the eligibility criteria were then invited to attend an introductory session with study personnel where the study and the AI model were described, and training on how to use the tool was administered.

Participating physicians informed their patients with MDD about the study and referred interested patients to the study personnel. Eligible patients were patients of enrolled physicians who were aged at least 18 years and diagnosed with MDD by the physician as per *Diagnostic and Statistical Manual of Mental Disorders, Fifth Edition* (*DSM-5*) criteria [19], able and willing to provide informed consent, and not diagnosed (or suspected) with bipolar affective disorder, as per DSM-5 criteria. Patients were required to be physically and mentally able to use a computer or smartphone (ie, to not be delirious or have sufficient cognitive function) but not necessarily to already be adept at it. Training was offered to patients who were not familiar with computer or mobile apps, and site-based computing resources were available to patients who did not have their own device; however, no patients required access to either these computing resources or training. Informed consent was obtained from patients, after which their account was created and linked to that of their physician. Patients accessed the web-based app via their own desktop or mobile devices; they were able to access the app in the setting of their choice, including at home or when they met their clinician. Patients and clinicians were compensated for their time. Research assistants were available for clinicians and patients as needed to provide technical support and provided patients with a training session on the app after account creation.

### Procedure

Upon account creation, patients were asked to complete the following questionnaires on the tool: Patient Health Questionnaire-9 (PHQ-9) to screen and track for depressive symptoms and their severity over time [20], General Anxiety Disorder-7 (GAD-7) to screen and track for anxiety symptoms and their severity over time [21], Alcohol Use Disorders Identification Test to screen for harmful alcohol use [22], Drug Abuse Screen Test to screen for the presence and severity of problematic drug use [23], and Standardized Assessment of Personality–Abbreviated Scale Self-Assessment to screen for personality disorders using a threshold of 3 points [24]. The results of the patient baseline questionnaire scores are summarized in Table 1. Patients identified other clinical comorbidities, such as migraines (2/14, 14% of patients) and anxiety disorders (7/14, 50% of patients), which are described in detail in Multimedia Appendix 1 [10,11,20,25-32], Table S2.

Patients were notified weekly by an automated email sent by the app to complete the PHQ-9; GAD-7; Patient Rated Inventory of Side Effects (to screen and track for specific antidepressant side effects and their severity); and Frequency, Intensity, Burden of Side Effects Rating (to assess the overall impact of antidepressant side effects) questionnaires [25].

Between obtaining informed consent and their next visit with their physician, patients met with study personnel to complete a demographic questionnaire (Table 2), Adverse Childhood Experiences questionnaire, and Life Events Checklist for DSM-5 (to screen for childhood or lifetime trauma [33,34]).

After enrollment into the study, patients had to complete a minimum of 3 visits: a baseline visit where their clinician did not use the tool, followed by at least 2 visits where the tool was used and appointment length was measured. These visits were intended to occur within a 4-month period per patient, with additional visits between or beyond visits 1 and 2 allowed and initially planned where the tool could be used; the visit frequency was intended to be at least monthly. However, as will be discussed, the impact of COVID-19 had a destabilizing effect on medical practices such that some patients experienced interappointment times that were longer than expected, leading to most patients completing only baseline, visit 1, and visit 2. However, this did not have an impact on the outcome, as only 2 postbaseline appointment lengths were intended to be measured. Patients were considered to have completed the study if they attended the baseline and visit 1 and 2 appointments at a minimum; completion of study-related tasks was not a criterion for study completion, given that this was a feasibility study focused on determining what patients would realistically complete. Research personnel recorded whether the baseline visit was an intake visit or a follow-up visit; this was relevant as initial intake visits are generally longer than follow-ups, and tracking this allowed for the adjustment of analyses such that initial intake visits would not artificially inflate the visit length at baseline. In the week preceding visit 1, patients completed the Quick Inventory of Depressive Symptomatology and met with study personnel to be administered the Inventory of Depressive Symptomatology, Clinician Rating [26,27]. These questionnaires were part of the set of questions used to generate the AI results.

Owing to COVID-19 and the public health recommendations released by the Québec government in March 2020, the study was adapted to be completed entirely from a distance. Originally, the protocol intended for appointment length to be recorded by research personnel on site, measured from the moment the patient entered the room to when they exited. However, because of the transition from in-person to telemedicine appointments (phone and video call), appointment length was measured as the length of the phone or video call during which the visit took place, as displayed on the physician or patient device and relayed verbally to research personnel. Further information about adaptation to COVID-19 can be found in the section *Telemedicine during COVID-19* in Multimedia Appendix 1.

After each visit where the tool was used, physicians were asked to complete a postappointment questionnaire describing device usability and any serious adverse events and to use the Udvalg for Kliniske Undersøgelser Side Effects Rating Scale [10,35] to record any side effects as perceived by the treating physician. Physician feedback was used to help identify and fix software errors (incorrect questionnaire score comparison in the CANMAT clinical algorithm noted at one visit and a broken link between pages in the CANMAT clinical algorithm noted at 2 back-to-back visits) and to refine the way the clinical algorithm presented the guidelines throughout the course of the study (by providing more context from the guideline paper for certain pieces of information, such as dose changes). Research personnel also administered the Brief Adherence Rating Scale to patients to estimate medication adherence since the prior visit [36].

Following visit 2, patients met with research personnel for end of study tasks, which consisted of completing the Quick Inventory of Depressive Symptomatology, the Scale to Assess Therapeutic Relationships in Community Mental Health Care-Patient (STAR-P) [37], a customized exit questionnaire designed specifically to capture elements of the experience of using this novel tool, as well as being administered the Inventory of Depressive Symptomatology–Clinician Rating and a custom semistructured interview. After all their patients completed the study, physicians were administered the Scale to Assess Therapeutic Relationships in Community Mental Health Care-Clinician (STAR-C), a customized exit questionnaire designed specifically to capture elements of the experience of using this novel tool, as well as a custom semistructured interview.

**Table 1:** Patient clinical baseline scores.

Questionnaire baseline score^a^			Patient, n (%)		Score, mean (SD)	
**SAPAS-SA^b^ (n=15)**						3.53 (2.23)
	<3 points: negative screen for personality disorder	5 (33)				
	≥3 points: positive screen for personality disorder. Further clinical evaluation is warranted.	10 (67)				
**GAD-7^c^ (n=14)**						12.21 (5.81)
	0-4: no or minimal anxiety	1 (7)				
	5-9: mild anxiety	4 (29)				
	10-14: moderate anxiety	3 (21)				
	15-21: severe anxiety	6 (43)				
**PHQ-9^d^ (n=15)**						14.80 (5.61)
	0-4: minimal or no depression	1 (7)				
	5-9: mild depression	1(7)				
	10-14: moderate depression	4(27)				
	15-19: moderately severe depression	7(47)				
	20-27: severe depression	2(13)				
**AUDIT^e^ (n=15)**						4.40 (3.58)
	<8: negative screen for harmful alcohol use	12 (80)				
	≥8: positive screen for harmful alcohol use	3 (20)				
**DAST-10^f^ (n=15)**						2.40 (3.60)
	0: no problems reported	8 (53)				
	1-2: low level	3 (20)				
	3-5: moderate level	0 (0)				
	6-8: substantial level	3 (20)				
	9-10: severe level	1 (7)				
WHODAS^g^ (n=13)			N/A		54.62 (27.08)	
**QIDS^h^ (n=12)**						13.12 (6.04)
	1-5: no depression	2 (17)				
	6-10: mild depression	2 (17)				
	11-15: moderate depression	3 (25)				
	16-20: severe depression	4 (33)				
	21-27: very severe depression	1 (8)				
**IDS-C^i^ (n=13)**						30.00 (12.88)
	0-11: no depression	0 (0)				
	12-23: mild depression	4 (31)				
	24-36: moderate depression	5 (38)				
	37-46: severe depression	3 (23)				
	47-84: very severe depression	1 (8)				
**ACE^j^ (n=13)**						1.62 (1.45)
	0	4 (31)				
	1	3 (23)				
	2	1 (8)				
	3	4 (31)				
	>4	1 (8)				

^a^Life Events Checklist for Diagnostic and Statistical Manual of Mental Disorders-5 questionnaire results can be found in Multimedia Appendix 1.

^b^SAPAS-SA: Standardized Assessment of Personality–Abbreviated Scale Self-Assessment.

^c^GAD-7: General Anxiety Disorder-7.

^d^PHQ-9: Patient Health Questionnaire-9.

^e^AUDIT: Alcohol Use Disorders Identification Test.

^f^DAST-10: Drug Abuse Screen Test-10.

^g^WHODAS: World Health Organization Disability Assessment Schedule.

^h^QIDS: Quick Inventory of Depressive Symptomatology.

^i^IDS-C: Inventory of Depressive Symptomatology, Clinician Rating.

^j^ACE: adverse childhood experiences.

**Table 2:** Patient demographics^a^.

Patient characteristics		Values
Age (years; n=14), mean (SD)		36.43 (14.84)
**Gender (n=14), n (%)**		
	Men	5 (36)
	Women	9 (64)
**Ethnicity (n=13), n (%)**		
	White	10 (77)
	Caribbean	1 (8)
	African or African American	1 (8)
	Unanswered	1 (8)
**Adoption status (n=13), n (%)**		
	Not adopted	12 (92)
	Adopted	1 (8)
**Residency status (n=13), n (%)**		
	Canadian citizen	11 (85)
	Immigrant status (>5 years ago)	1 (8)
	Immigrant status (<5 years ago)	1 (8)
**Relationship status (n=13), n (%)**		
	Married	4 (31)
	Divorced	1 (8)
	Dating a single partner	2 (15)
	Not in a relationship	6 (46)
**Employment status (n=13), n (%)**		
	Full time	7 (54)
	Part time	1 (8)
	Disability (not working)	2 (15)
	Unemployed and volunteer work	2 (15)
	Unemployed	1 (8)
**Highest level of education (n=12), n (%)**		
	Master's degree	3 (25)
	Bachelor’s degree	1 (8)
	University, no degree	3 (25)
	Collège d’enseignement général et professionnel	3 (25)
	High school or equivalent (GED^b^)	2 (17)
Years of education (n=12), mean (SD)		15 (6.42)
Income (n=10, Can $; US $), mean (SD)		82,333.33 (70,099.93); 64,829.39 (55,196.80)

^a^14 patients completed the study; however, 1 patient did not complete the demographic questionnaire.

^b^GED: General Educational Development.

### Analyses

#### Assessing the Feasibility of the CDSS for Use in Clinical Practice

Appointment lengths at baseline, visit 1, and visit 2 were extracted from the research assistant appointment log and analyzed using the SPSS (version 27) repeated measures analysis of variance (ANOVA; 3 factors, within-subject variables). Although complete appointment length data were available for 14 patients, one of the patients’ baseline appointments could not be counted as the physician opened the tool to look at questionnaire answers and input current patient medications. Data from 13 patients were included in the final repeated measures ANOVA, with their means and SDs reported below. Descriptive data about the subjective view of appointment length were also extracted from the custom exit questionnaires.

To determine the validity of our sample with respect to the main outcome of appointment length, we conducted 2 sample size calculations using the G*Power package, version 3.1.9.7 [38]. The first was a sample size calculation for a repeated measures ANOVA with 1 group, three measures, a moderate effect size estimate (0.25), an α of .05, 80% power, and a correlation between responses of 0.5. This was intended to determine the power required to detect within-group differences over time. The calculated required sample size was 28, validating our target sample size of 30-40 patients. Our second sample size estimation was based on the need to detect a clinically significant increase in time, which was set as being 5 minutes based on physician comments in a previous study [10]. Here, we calculated the sample required to detect an increase of at least 5 minutes (ie, target sample mean of 25, SD 5 minutes) compared with a standard 20-minute interview (SD is an estimate based on a reasonably expected variation in appointment length). The α was set at .05 and power at 80%, resulting in a required sample size of 8 patients.

#### Assessing Physician and Patient Trust in the CDSS and Its Effect on the Clinician-Patient Relationship

Descriptive data about physician and patient trust in the CDSS were extracted from custom exit questionnaires. The physician-patient relationship was assessed by examining STAR-P and STAR-C scores, as well as patient ratings of the relationship extracted from the custom exit questionnaires.

#### Assessing the Usability of the CDSS

Descriptive data concerning physician and patient ratings of CDSS ease of use were extracted from custom exit questionnaires.

#### Assessing Engagement With the App

Physician engagement was assessed by determining the percentage of study visit days where the app was opened by the physician; this was determined by checking physician log-in dates and their correspondence to patient appointment dates. Patient engagement was assessed by measuring the percentage of questionnaires actually sent that were completed each week for PHQ-9 and GAD-7. Questionnaire completion was measured from the date of account creation (week 1 in the study) to week 12, a timeframe chosen to concord with the 12-week follow-up time planned for our upcoming clinical trial. For other timeframes, see Multimedia Appendix 1. The number of PHQ-9 questionnaires that were completed in the app by patients was calculated by subtracting those completed by physicians and taking the mean completion rate across all patients in each of the three time intervals. Note that only the PHQ-9 could be completed by physicians; all other questionnaires in the CDSS could be completed by patients only.

## Results

### Recruitment and Safety Data

**Figure 1:**
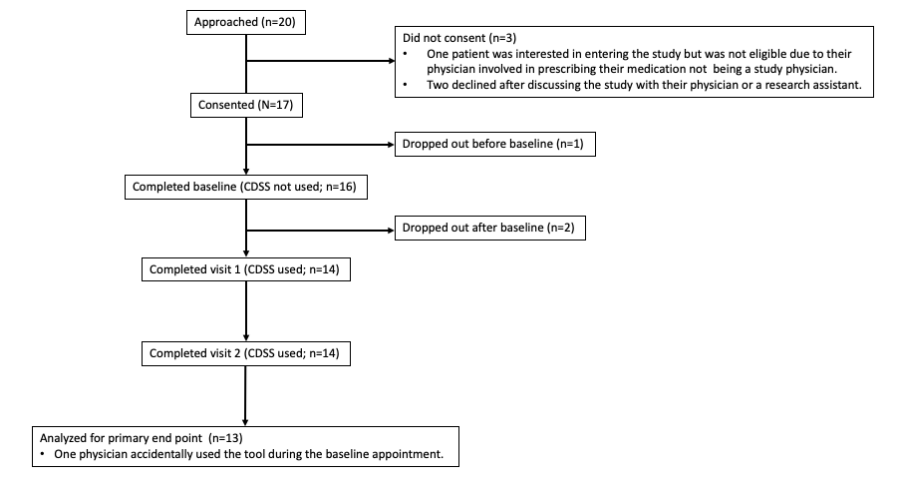
Consolidated Standards of Reporting Trials study flow chart of participant recruitment and completion. CDSS: clinical decision support system.

A total of 10 physicians were initially recruited; however, of the 10 physicians, 3 (30%) psychiatrists were unable to recruit patients because of COVID-19 related interruptions in regular clinical practice and could not be included (of the 3 psychiatrists, 2 [67%] of the psychiatrists’ day programs were closed, and 1 [33%] of the psychiatrist focused on providing consults rather than follow-up appointments during the pandemic). A total of 20 patients were approached by the 10 physicians recruited for the study (Figure 1) [39]. Of these 20 patients, 2 (10%) declined participation after discussing the study with their physician or a research assistant. One patient who was interested in entering the study was not eligible as another physician involved in prescribing their medication was not a study physician and as such would not be able to use the app to follow the patient. The recruiting physician was running a day hospital program, which the patient in question was attending. Of the 20 patients, 17 (85%) patients were recruited into the study. As such, 85% (17/20) of the patients approached were recruited. Of the 17 patients, 14 (82%; Table 2) completed the study (defined as attending baseline, visit 1, and visit 2 appointments). One patient withdrew before the baseline appointment, and 2 withdrew after the baseline appointment but before the CDSS was used at visit 1. Of the 14 patients, the sample of patients completing the study consisted of 9 (64%) women and 5 (36%) men with a mean age of 36.43 years (SD 14.84). See Table 2 for demographics and Table 1 for the baseline questionnaire scores. The pandemic was a reason for significantly reduced recruitment and for the withdrawal of several patients from the study.

Of the 17 patients, 2 (12%) experienced side effects, as recorded on the Udvalg for Kliniske Undersøgelser Side Effects Rating Scale across the course of the study (see adverse events in Multimedia Appendix 1 for more details). Of the 17 patients, 1 (6%) discontinued treatment. We noted that discontinuation rates of psychiatric treatment could often be >40% [40]. There were no serious adverse events related to the tool; however, of 17 patients, 1 (6%) experienced 2 emergency room visits (a work-related injury and a rash that was thought to be viral by consultants in the emergency room; however, this may have been related to a new antidepressant prescription that was made by a physician without reference to the AI predictions) during the study.

### Assessing the Feasibility of the CDSS for Use in Clinical Practice

**Figure 2:**
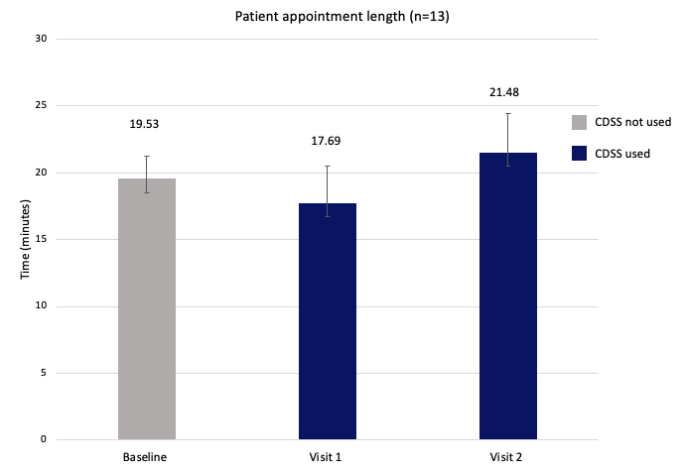
Patient appointment times. A total of 14 patients completed the study; however, 1 patient’s appointment time could not be counted at baseline because of their physician opening the clinical decision support system erroneously. CDSS: clinical decision support system.

Patients (n=14) were in the study for an average of 13.2 weeks or 92.4 days (SD 9.74 weeks or SD 68.18 days), excluding 2 patients who dropped out of the study within 1 week of creating their accounts. The mean time between the baseline appointment and visit 1 was 40.86 days (SD 29.40), and the mean time between visit 1 and visit 2 was 51.57 days (SD 62.58). For the 13 patients for whom the appointment length analysis was carried out, baseline visits lasted a mean of 19.53 minutes (SD 6.09). Visit 1 and visit 2 lasted a mean of 17.69 minutes (SD 10.12) and 21.48 minutes (SD 10.69), respectively (Figure 2). Our findings showed no significant difference between the baseline appointment time without the CDSS and subsequent visits using the CDSS (*F*_2,24_=0.805; mean square error=58.08; *P*=.46).

With regard to the subjective physician view of appointment length, of the 7 physicians, 4 (57%) rated that using the tool “took about the same time as my usual practice,” indicated by a rating of 3 on a 5-point Likert scale. In addition, of 13 patients, 8 (62%) felt that their appointment time did not change, whereas 1 (8%) patient felt that it decreased.

### Assessing Physician and Patient Trust in the CDSS and Its Effect on the Clinician-Patient Relationship

With regard to the tool’s trustworthiness, 62% (8/13) of patients and 71% (5/7) of physicians rated that they trusted the CDSS, indicated by a 4 or 5 on a 5-point Likert scale. The mean STAR-P and STAR-C scores were 42.69 (SD 5.57) and 40.29 (SD 5.65), comparable with 38.4 (SD 12.0) and 31.5 (SD 6.9) in the original Scale to Assess Therapeutic Relationships in Community Mental Health Care study [37], respectively, indicating no major negative effects of the CDSS on the clinician-patient relationship (a possible outcome, given we included a new piece of technology that was directly involved in the shared decision-making process). Further information about the Scale to Assess Therapeutic Relationships in Community Mental Health Care subscales is present in Multimedia Appendix 1. In addition, on their custom exit questionnaire, 46% (6/13) of patients felt that the patient-clinician relationship significantly or somewhat improved, whereas 54% (7/13) of patients felt that it did not change.

### Assessing the Usability of the CDSS

Good overall usability of the CDSS, indicated by a 4 or 5 on a 5-point Likert scale, was rated by 92% (12/13) of patients and 71% (5/7) of physicians (Multimedia Appendix 1, Table S3).

### Assessing Engagement With the App

At each patient’s visit 1, all (7/7, 100%) physicians logged into the tool on the same day as the visit, and the clinical algorithm module (which contains the CANMAT guidelines and AI results, when available) portion of the tool was accessed on 93% (13/14) of appointment days. At the subsequent visit 2 appointments, once again, all (7/7, 100%) physicians logged into the tool, whereas the clinical algorithm component was again accessed at 93% (13/14) of appointments.

**Figure 3:**
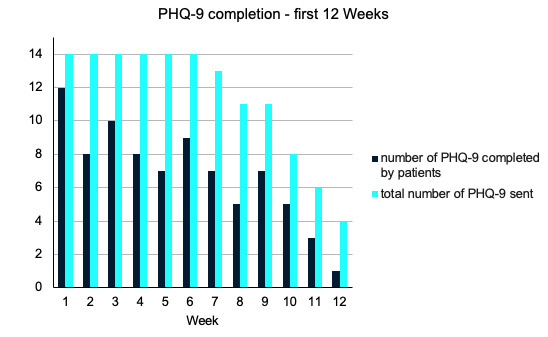
Frequency of PHQ-9 completion by patients in the first 12 weeks of the study versus the total number sent in the clinical decision support systems (1 per week, per active patient). PHQ-9: Patient Health Questionnaire-9.

**Figure 4:**
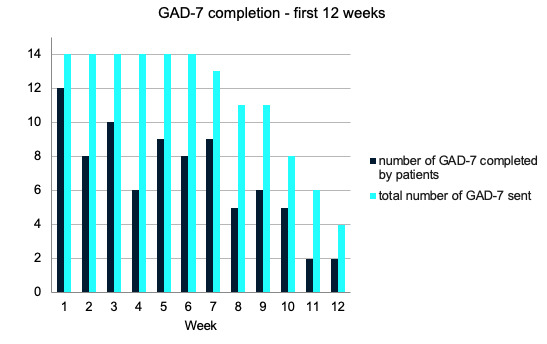
Frequency of GAD-7 questionnaires completed by patients in the first 12 weeks of the study versus the total number sent in the clinical decision support systems (1 per week, per active patient). GAD-7: General Anxiety Disorder-7.

**Figure 5:**
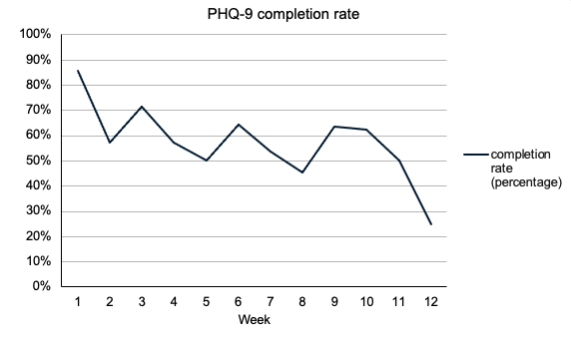
Percent of PHQ-9 completion by patients in the clinical decision support systems during the first 12 weeks of the study. PHQ-9: Patient Health Questionnaire-9.

**Figure 6:**
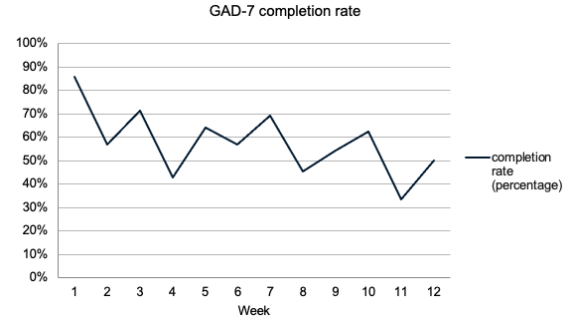
Percent of GAD-7 questionnaires completed by patients in the clinical decision support systems during the first 12 weeks of the study. GAD-7: General Anxiety Disorder-7.

Figures 3-6 demonstrate the PHQ-9 and GAD-7 completion rates each week during the first 12 weeks of the study. The light bars in Figures 3 and 4 reflect the total number of questionnaires sent, given the number of patients that were active in the study during weeks 1 through 12. The total number of PHQ-9 and GAD-7 questionnaires completed by patients on the app for the first 12 weeks of the study were summed and are shown in the dark bars in Figures 3 and 4. In each of weeks 4, 5, 6, and 10, 1 patient completed their PHQ-9 questionnaire with a physician. For each of these weeks, one response was subtracted from the total number of PHQ-9 questionnaires completed to reflect only those done by patients (Figures 3 and 5).

The mean completion rate of all PHQ-9 questionnaires sent from account creation to week 12 of the study was 64% (SD 28%). The mean completion rate of the PHQ-9 by patients alone in this time frame was 59% (SD 28%). The GAD-7 had a mean completion rate of 60% (SD 28%) in the first 12 weeks of the study. Completion rates for other time frames can be found in Multimedia Appendix 1 but were similar.

Of the 14 participants, 10 (71%) had mean PHQ-9 and GAD-7 completion rates ≥50%. The lowest completion rate among the patients for both questionnaires sent in the first 12 weeks was 8%. Most patients regularly completed questionnaires, with 76% (11/14) completing ≥33% of app assessments and 71% (10/14) completing ≥50% of the PHQ-9 and GAD-7 assessments in the app over the first 12 weeks in the study.

Biweekly peaks in the completion rates were observed for the PHQ-9 and GAD-7 questionnaires (Figures 3 and 4). Although they were intended to be completed weekly as a part of the study, these questionnaires are often administered at ≥2-week intervals in practice, indicating the likely feasibility of a reduced questionnaire frequency.

In addition, an exploratory correlation analysis aimed at determining potential correlates of questionnaire completion rates revealed that patients who had appointments scheduled further apart were less likely to complete the PHQ-9 (*r*_12_=–0.69; *P*=.006).

Results and a full discussion of the changes in depression and anxiety questionnaire scores can be found in Multimedia Appendix 1.

High rates of treatment adherence were noted as measured by the Brief Adherence Rating Scale and can be found in Multimedia Appendix 1.

## Discussion

### Principal Findings

The primary objectives of the study were to assess feasibility, usability, and ongoing engagement with the CDSS when integrated into clinical practice, as well as to measure physician and patient trust in the CDSS and its impact on the clinician-patient relationship. The primary feasibility outcome of interest was appointment length to determine whether the use of the CDSS required more time than the baseline appointment. We were able to confirm that appointment length did not significantly increase after the introduction of the tool. In addition, patients and clinicians provided high trust and usability ratings; the app resulted in improved patient-clinician relationship for some patients, and patients and clinicians both engaged with the app in a manner consistent that supports clinical feasibility. Digital application use for the purpose of promoting mental health is increasingly recommended by public health organizations. For example, in its Mental Health Action Plan 2013-2020, the World Health Organization proposed “the promotion of self-care, for instance, through the use of electronic and mobile health technologies” [41]. Meanwhile, the UK National Health Service’s website endorses a short list of web-based mental health resources, which includes smartphone-based apps [41]. Specifically, studies on app usability in the treatment of depression have demonstrated that telemedicine and internet-based approaches are feasible and as effective as in-person treatment [42].

However, these tools also face substantial barriers with regard to adherence. For example, a randomized clinical field trial conducted by Arean et al [42] compared 3 different mobile apps for depression to examine how individuals who download these tools typically use them. The authors’ findings show that most participants did not use their assigned intervention apps as instructed and experienced a significant drop off in use after 2 weeks [42]. In addition, a study that investigated the feasibility of using a smartphone app to assess schizophrenia prompted patients via SMS text messages to complete personalized questionnaires once per week. They found that participants (n=18) completed 65% of app assessments, “with 78% completing ≥33% app assessments and 72% completing ≥50% app assessments” [43], similar to the results observed in this study. In summary, response rates observed in our study (with 10/14, 71% of patients completing at least 50% of assessments) were reasonable in the context of previous reports, and engagement persisted fairly stably beyond 2 weeks, demonstrating that the app was able to retain patients at least as much or more consistently than applications in previous studies. In addition, physician engagement was high, with physicians opening the tool at each visit.

Physician engagement with mental health apps is related to their technological competency, their perception of patient access to technology, and organizational infrastructure that facilitates the adoption of the apps into their practice [44], which are all factors considered when designing the study; for example, physicians who were less technically oriented could rely on study staff to provide ongoing support for app use as needed. The results of our study demonstrate sustained patient and physician engagement beyond 2 weeks, potentially because the app was directly tied to clinical care and because high physician use of the app and the data patients inputted may have motivated patients to continue engaging. Indeed, higher rates of engagement are linked to the use of telephone and email reminders, as well as follow-up with a physician [45], a finding supported by our demonstration of lower PHQ-9 response rates as a result of longer interappointment lengths. In addition, the email reminders sent to patients to complete assessments likely had a positive impact on completion rates based on these previous findings.

More than half (4/7, 57%) of the physicians felt that using the tool in session took approximately the same time as their usual practice. A systematic literature review conducted by Kerst et al [46] found that 70.2% of physicians treating depression had never used applications in clinical practice before, suggesting that the integration of mental health tools remains quite novel. Therefore, it is possible that some physicians reported that their appointments felt longer than their usual practice simply because they were not yet familiar with the tool. Interestingly, most patients did not subjectively report that the tool increased their appointment time. Objectively, appointment length did not significantly increase when the tool was introduced, lending credence to the idea that the novelty of the tool use may have influenced the perception of time spent by physicians.

We found that 62% (8/13) of patients endorsed some degree of trust in the CDSS, somewhat lower than the percentage of clinicians (5/7, 71%) with some degree of trust. This may have in part been because of COVID-19: most clinicians followed up with their patients by phone, which meant that patients did not get to view the AI results on the physician’s screen as intended, which may have improved feelings of trust had it occurred more frequently; standardized patients noted that looking at the screen with their physicians was a positive experience in our previous simulation center study [10]. Nonetheless, patients’ mean score was 3.85 on a 5-point Likert scale, which indicates that patient trust trends in a positive direction.

### Limitations

The main limitation of the study is the small sample size, which limits the strength of the conclusions that can be drawn from this study. All results should be considered preliminary and are only intended to demonstrate feasibility; similar metrics of feasibility and ease of use will be used during our upcoming clinical trial to confirm and expand upon these findings. Another weakness is the heterogeneity in the severity of the patients’ depression, which limits our ability to comment on tool effectiveness and generalize these results to different strata of illness severity. Nevertheless, it also presents as a strength because it allowed us to demonstrate feasibility in a range of clinical situations.

Our study fell short of recruitment targets, which may have been in part because of several scenarios. One possibility is that there were sufficient patients available to recruit but that these patients were not interested in participating in the study. However, this is not supported by the high proportion of approached patients who agreed to participate. Furthermore, of the patients who did participate, dropout rates were comparable with other studies of mental health applications, as reviewed in the introduction. Another more likely scenario is that there were fewer patients available to recruit than expected; this seems to have been the case because of the COVID-19 pandemic. As discussed, 3 of our physicians were unable to recruit patients because their practices were significantly changed by the pandemic, and they either did not follow patients longitudinally or had very few new patients who were eligible to participate. In addition, physicians who successfully recruited patients frequently commented on the lack of patients presenting with MDD (although this was not formally recorded). As such, the low sample size in this study is not indicative of patient interest in participation but rather of the number of patients available to be recruited during the pandemic. We noted that in unpublished data from a quality improvement project, which included a control group using a version of the CDSS without the AI enabled (ie, providing measurement-based care and algorithm-guided treatment but no AI predictions), which was initiated later in the pandemic (once clinical practices had returned to some stability), recruitment speed was much faster, with 34 patients recruited in 7 months, indicating that recruitment should not be a barrier for our upcoming clinical trial.

However, the low sample size does mean that our study was underpowered to detect differences in appointment length within groups over time when considering an a priori sample size calculation for repeated measures as described above. Despite this, there are 2 reasons why this study provides useful information. First, the appointment lengths are interpretable data. Should appointment lengths have been different in the order of ≥5 minutes, which in previous work [10] has been identified by clinicians as the amount of time they would be willing to spend on the CDSS, and had this difference not been significant simply because of low power, then we would have to remain concerned that the tool might increase appointment lengths. However, the recorded appointment lengths differed by <5 minutes; even if this difference had been statistically significant, it would not have been clinically significant. Indeed, in the sample size calculation discussed earlier, where the objective was to detect an appointment length of at least five minutes more than a usual 20-minute length appointment, the required sample size was only 8, meaning that our study should have been sufficiently powered to detect this difference from a standard appointment length (which the baseline sessions matched closely). In addition, in the unpublished quality improvement project mentioned above, appointment lengths in the active group, which included 22 patients who used the tool, were also roughly 20 minutes long. Finally, as noted, most patients and physicians did not note a subjective increase in the appointment length. As such, although the study may be underpowered, there does not seem to be a signal in the available data to suggest a clinically significant increase in appointment length, which would reduce feasibility.

An additional and significant limitation of this study is that although its design allowed us to examine the impact of introducing the tool on the patient-clinician relationship and the clinician workflow, this at the same time prevented us from examining the effectiveness of the device in terms of improvement in depression scores. This is because the tool being introduced well into a patient’s treatment course could not have its intended effect of assisting treatment selection or helping clinicians implement measurement-based care and algorithm-guided treatment across the entire length of the study. This was compounded by the delays between appointments and the reduced number of visits as a result of COVID-19. However, we note that the decision was made during study design not to focus on effectiveness and, because of the novelty of the device and the need to determine challenges to its introduction into clinical practice, to focus squarely on feasibility. As such, the modest improvements in depression and anxiety scores seen here are in line with expectations, given that the tool was not introduced in a manner where it could have its intended effect on patient care.

With regard to feasibility, ease of use, and the ability to correct any major limitations, it would be reasonable to be concerned about whether this study, with its small sample size, could on its own speak to the readiness of the tool for clinical trials. However, this study should be considered in the context of previously published evidence on the same tool, which demonstrated its ease of use in a simulation center environment [10]. In addition, several pilot projects and quality improvement projects have been undertaken with the non-AI version of this device. These pilots, although not undertaken as research projects, allowed for the testing of the user interface and the incorporation of user feedback and were conducted alongside intensive quality assurance in the development process. The purpose of this study was to test this tool with the AI enabled in the context of longitudinal follow-up. The present results in this context, combined with the adjustments and corrections that have been made through quality assurance, physician feedback in this study, and other pilot projects, allow us to conclude that the tool is ready for clinical trials.

### Conclusions

In this paper, we have demonstrated that the Aifred CDSS is feasible and easy for clinicians and patients to use in a longitudinal manner and that it does not require increased time to use in clinic. In addition, the tool had an interesting impact on the clinician-patient relationship. For roughly half of the patients, it did not negatively or positively affect the relationship, helping to allay concerns about technological solutions worsening relationships between clinicians and patients. For the other half of the patients, the relationship was actually rated as having improved, indicating that for some patients, the CDSS may have beneficial effects on the clinician-patient relationship. This latter point will be further elaborated in a future study and should be investigated in future work. Planned clinical trials will serve as an opportunity to confirm these feasibility results and to determine if the CDSS is effective in improving depression outcomes.

## Appendix

Multimedia Appendix 1 Peer-reviewed supplementary material.

## Abbreviations

AI: artificial intelligence
ANOVA: analysis of variance
CANMAT: Canadian Network for Mood and Anxiety Treatments
CDSS: clinical decision support system
*DSM-5*: *Diagnostic and Statistical Manual of Mental Disorders, Fifth Edition*
GAD-7: General Anxiety Disorder-7
MDD: major depressive disorder
PHQ-9: Patient Health Questionnaire-9
STAR-C: Scale to Assess Therapeutic Relationships in Community Mental Health Care-Clinician
STAR-P: Scale to Assess Therapeutic Relationships in Community Mental Health Care-Patient
