# Correction: Evaluating the Clinical Feasibility of an Artificial Intelligence–Powered, Web-Based Clinical Decision Support System for the Treatment of Depression in Adults: Longitudinal Feasibility Study

**DOI:** 10.2196/56570

**Published:** 2024-01-24

**Authors:** Christina Popescu, Grace Golden, David Benrimoh, Myriam Tanguay-Sela, Dominique Slowey, Eryn Lundrigan, Jérôme Williams, Bennet Desormeau, Divyesh Kardani, Tamara Perez, Colleen Rollins, Sonia Israel, Kelly Perlman, Caitrin Armstrong, Jacob Baxter, Kate Whitmore, Marie-Jeanne Fradette, Kaelan Felcarek-Hope, Ghassen Soufi, Robert Fratila, Joseph Mehltretter, Karl Looper, Warren Steiner, Soham Rej, Jordan F Karp, Katherine Heller, Sagar V Parikh, Rebecca McGuire-Snieckus, Manuela Ferrari, Howard Margolese, Gustavo Turecki

**Affiliations:** 1 Aifred Health Inc. Montreal, QC Canada; 2 University of Waterloo Waterloo, ON Canada; 3 McGill University Montreal, QC Canada; 4 University of Cambridge London United Kingdom; 5 University of Arizona Tucson, AZ United States; 6 Duke University Durham, NC United States; 7 University of Michigan Ann Arbor, MI United States; 8 Barts and the London School of Medicine London United Kingdom; 9 Douglas Mental Health University Institute McGill University Montreal, QC Canada

In “Evaluating the Clinical Feasibility of an Artificial Intelligence–Powered, Web-Based Clinical Decision Support System for the Treatment of Depression in Adults: Longitudinal Feasibility Study” (JMIR Form Res 2021;5(10):e31862), the authors noted one error.

In the section “Assessing Physician and Patient Trust in the CDSS and Its Effect on the Clinician-Patient Relationship”, the STAR-P and STAR-C total scores were listed as:

The mean STAR-P and STAR-C scores were 33.62 (SD 2.90) and 31.14 (SD 2.63) comparable with 38.4 (SD 12.0) and 31.5 (SD 6.9) in the original Scale to Assess Therapeutic Relationships in Community Mental Health Care study.

They have been changed to read in the following manner:

The mean STAR-P and STAR-C scores were 42.69 (SD 5.57) and 40.29 (SD 5.65), comparable with 38.4 (SD 12.0) and 31.5 (SD 6.9) in the original Scale to Assess Therapeutic Relationships in Community Mental Health Care study.

The correction will appear in the online version of the paper on the JMIR Publications website on January 24, 2024 together with the publication of this correction notice. Because this was made after submission to PubMed, PubMed Central, and other full-text repositories, the corrected article has also been resubmitted to those repositories.

